# Application of a Cone-Beam Computed Tomography-Based Index for Evaluating Surgical Sites Prior to Sinus Lift Procedures—A Pilot Study

**DOI:** 10.1155/2021/9601968

**Published:** 2021-12-31

**Authors:** Shishir Ram Shetty, Satyavrat Arya, Vinayak Kamath, Saad Al-Bayatti, Hesham Marei, Hossam Abdelmagyd, Mohamed El-Kishawi, Saaid Al Shehadat, Sausan Al Kawas, Raghavendra Shetty

**Affiliations:** ^1^Department of Oral and Craniofacial Health Sciences, College of Dental Medicine, University of Sharjah, UAE; ^2^Medanta, The Medicity, Gurugram, India; ^3^Department of Public Health Dentistry, Goa Dental College and Hospital, Bambolim, Goa, India; ^4^College of Dentistry, Gulf Medical University, Ajman, UAE; ^5^Department of Preventive and Restorative Dentistry, College of Dental Medicine, University of Sharjah, UAE; ^6^Department of Clinical Sciences, College of Dentistry, Ajman University, UAE

## Abstract

**Objectives:**

Radiography-based indices can help surgeons perform detailed examinations of the surgical site and predict the surgical difficulty of cases. We aimed to develop and validate a novel CBCT-based index that can predict the surgical difficulty of sinus-augmentation procedures.

**Materials and Methods:**

In the first stage, five experienced dental specialists performed a review of the literature and closed group discussions and designed the novel index. In the next stage, the index was validated. CBCT scans of 30 patients scheduled for sinus-augmentation procedures were evaluated and assigned presurgical CBCT evaluation scores (PSCESs) by five examiners. Subsequently, one oral surgeon performed sinus augmentation using the lateral antrostomy technique and assigned surgical difficulty scores (SDSs) to each of the 30 cases along with 2 observers. The PSCESs and SDSs were statistically analysed to determine the interrater reliability and validity of the index.

**Results:**

The interrater agreement of the PSCES among the five presurgical evaluators was 0.85. The PSCES of the five evaluators had highly significant correlation (*P* < 0.001, *r* = 0.68 to 0.76) with the SDS. Regression analysis revealed that for every unit increase in the PSCES, there is 0.46 to 0.57 increase in the SDS value.

**Conclusion:**

The results of this pilot study revealed that a novel CBCT-based index can be used as a reliable tool for predicting the surgical difficulty of sinus-augmentation procedures. However, the novel index needs to be tested on a larger sample of patients and evaluators for a more concrete validity and reliability.

## 1. Introduction

Dental implants have become an integral part of clinical practice, and the number of dental implant placements is estimated to substantially exceed the number of artificial hip and knee joint replacements in the United States of America annually [1]. Nevertheless, placement of implants in the posterior edentulous maxilla remains a challenging task due to the anatomical complexities of the maxillary sinus [2]. Therefore, accurate assessment of the maxillary sinus is important in surgical and implant procedures involving the maxillary posterior region [3]. Sinus augmentation may be required in certain situations when the alveolar bone height is not adequate for implant placement. Sinus augmentation can be performed using two different techniques [4]. In the direct method, access is gained through lateral antrostomy. In the indirect method, the approach is through the alveolar crest [4]. Certain key anatomical factors can determine the difficulty in sinus-augmentation techniques, and some of these factors can be evaluated with a cone-beam computerized tomography (CBCT) scan prior to the surgical procedure [5]. One of the important factors is the thickness of the lateral wall of the maxillary sinus, which appears to influence the intactness of the sinus membrane [6]. During the osteotomy procedure in the lateral wall, the sinus membrane may tear if excessive pressure is exerted on a thin wall [7]. The second important factor is the nature of the sinus membrane [8]. The presence of sinus septations and sinus pathology also needs to be considered during the augmentation procedure [8]. Sinus-related diseases and abnormalities are found in approximately 40% of the patients listed for sinus-augmentation procedures [9]. The presence of pathologies hinders the surgical procedures and predisposes the patient to postoperative complications [10, 11]. Several such factors have to be evaluated thoroughly by the oral surgeon in CBCT images of the surgical sites prior to osteotomy procedures in the maxillary posterior edentulous region. Thus, there is a need to develop and validate a CBCT-based index that comprehensively focuses on all important anatomical structures and their variations in the surgical site. The aim of this study was to evaluate whether a novel CBCT-based index could predict the surgical difficulty encountered in maxillary sinus-augmentation procedures. The objectives of the study were to correlate the presurgical CBCT evaluation score (PSCES) with the surgical difficulty score (SDS) and to determine whether the PSCES could predict the SDS in sinus lift procedures. The null hypothesis of the study was that the novel CBCT-based index cannot predict the surgical difficulty encountered in sinus-augmentation procedures.

## 2. Methods

### 2.1. Study Design

A two-stage study was designed to develop and validate a CBCT-based index for evaluating the surgical site prior to sinus-augmentation procedures. Ethical approval was obtained from the Institutional Review Board of the Gulf Medical University, United Arab Emirates (Reference number INT/COD/FR/006-2020 dated 27th January 2020). All methods were performed in accordance with the ethical principles for medical research (Declaration of Helsinki, 1964 [12].

#### 2.1.1. Stage 1: Development of the Index

The team involved in designing the novel index included 3 oral surgeons and 2 oral radiologists. All members of the team had more than 10 years of clinical experience in their respective specialties. They held group discussion on the anatomical factors influencing the difficulty in sinus-augmentation procedures. Once an agreement was reached among the five team members, a final version of the index was prepared (Table 1).

**Figure 1:**
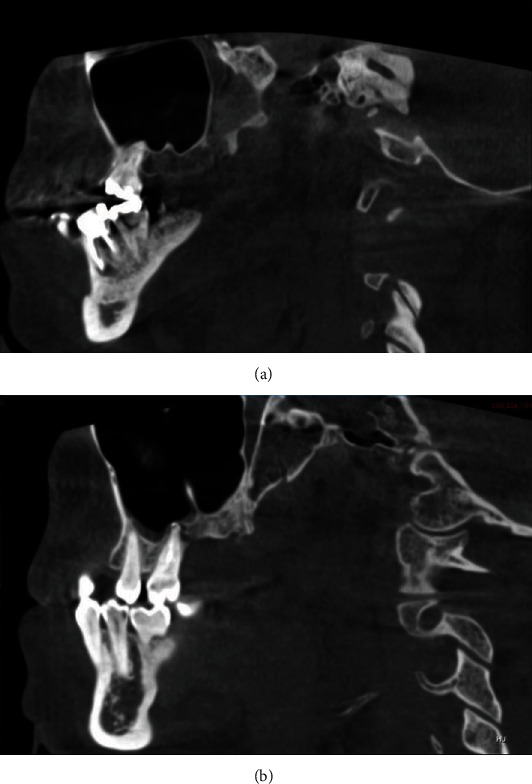
(a) Sagittal CBCT section showing presence of a small partial septation above surgical site. (b) Sagittal CBCT section showing large partial septation above surgical site.

**Figure 2:**
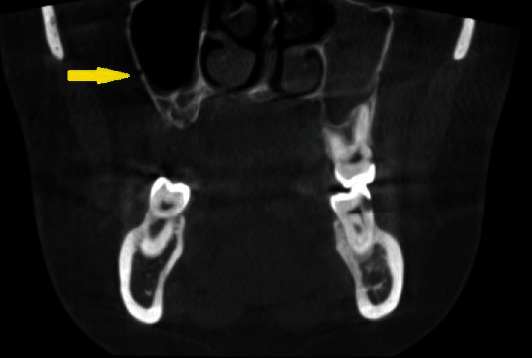
Lateral antral artery (yellow arrow) seen in the lateral wall of the right maxillary sinus.

**Figure 3:**
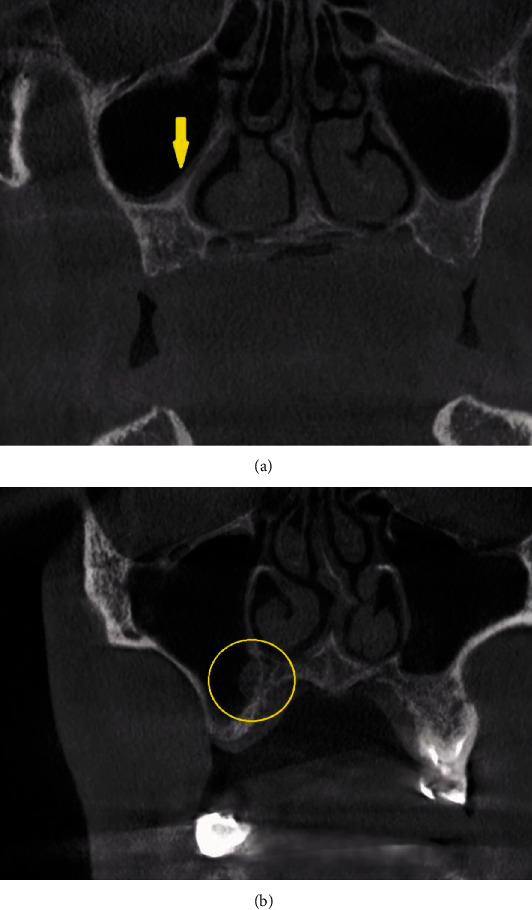
(a) Coronal CBCT section showing evidence of mucosal thickening (yellow arrow). (b) Coronal CBCT section showing mucous-retention cyst in the right maxillary sinus proximal to the osteotomy site (yellow circle).

The index included six key parameters: thickness of the lateral wall of the sinus, sinus septations (Figures 1(a) and 1(b)), presence of an alveolar antral artery (Figure 2), relationship of the sinus membrane with the roots of the adjacent teeth, thickness of the sinus membrane, and the presence of sinus pathologies (Figures 3(a) and 3(b)).

#### 2.1.2. Stage 2: Validation of the Index

To evaluate whether the index as the whole and the individual parameters truly reflected the clinical situation, we assigned scores for each of the six components of the index. The sum of the scores of the individual parameters was termed as the PSCES.

We conducted a pilot study by correlating PSCES and SDS in sinus augmentation among 5 patients. A moderate positive correlation (*r* = 0.5) was observed. On the basis of this correlation value, and the availability of the study subjects reporting to the hospital, a minimal sample size of 30 was considered adequate for the study. CBCT scans of 30 participants requiring sinus-augmentation procedures were evaluated using the novel index by 5 oral surgeons (SA, HM, ST, GT, and AK) with a minimum of 10 years of experience. The presurgical evaluators were not involved in developing the index). The mean age of the study participants was 47 years, and the age range was 27 to 66 years. Patients with specific sinus pathologies were excluded from the study based on the algorithm suggested by Friedland and Metsun in 2013 [13]. The specific sinus pathologies excluded from the study are listed below. Patients with these sinus pathologies were referred for further evaluation and treatment.

1. History of maxillary sinusitis within the past one year
2. Sinus membrane thickening greater than 2 mm
3. Mucous-retention cyst filling more than 75% of the sinus
4. Presence of air-fluid level in the sinus
5. Patent oroantral communication
6. Presence of teeth and other foreign bodies of the sinus
7. Missing sinus wall
8. Sinus polyps, benign, and malignant tumours.

The scans were obtained using a CBCT machine (ProMax 3D Mid; Planmeca, Helsinki, Finland). The CBCT machine was operated at 90 kVp and 10 mA with a 16 × 9 cm^2^ field of view, and the voxel size was 400 *μ*m. Assessment of CBCT scans was performed directly on a 1920 × 1080 pixel, 23-inch screen (DELL monitor; Dell, Round rock, TX, United States of America). The evaluators assigned PSCESs for each of the 30 patients. An average PSCES was calculated for each case. Based on the PSCES, the cases were classified into difficulty levels. The levels were minimally difficult (0 to1.9), moderately difficult (2 to 3.9), and difficult (4 to 6).

In the next step, one oral surgeon (performer), who was blinded to the PSCES and not involved in index formulation of the novel index, performed sinus-augmentation procedures. After completing the sinus-augmentation procedure, surgeon A assigned the difficulty score for the case based on the surgical difficulty score (SDS) checklist. The SDS checklist evaluated 4 intrasurgical parameters and one postsurgical parameter (Tables 2 and 3).

To avoid bias in scoring surgical difficulty, two surgeons (observer 1 and observer 2, who were blinded to the PSCES and not involved in index formulation of the novel index) observed the video recording of the surgery and independently assigned the SDSs. If any postoperative complications were reported during the follow-up visits (one month from the date of surgery), the information was shared among the three surgeons, and a score was assigned in the SDS checklist.

The data collected were entered into a Microsoft Excel spreadsheet and statistically analysed (IBM SPSS Version 22; International Business Machine, Armonk, NY, United States of America). The Fleiss kappa was used to assess the interrater agreement among CBCT evaluators and surgeons. *P* values < 0.05 were considered statistically significant.

## 3. Results

A good interrater agreement of the PSCES among the presurgical evaluators was 0.85 (Table 4).

Similarly, a very good interrater agreement of the SDS between the performer and observer 1 and observer 2 (0.82). There was a good interrater agreement between observer 1 and observer 2 (0.64) (Table 5).

The PSCES of the five evaluators had highly significant correlation (*P* < 0.001, *r* = 0.68 to 0.76) with the SDS of the performer (Table 6).

Regression analysis between the independent variable PSCES and dependent variable SDS revealed a highly significant (*P* < 0.001) positive association (Table 7).

The coefficient values ranged from 0.46 to 0.57, indicating that for every unit increase in the PSCES, there is 0.46 to 0.57 increase in the SDS value.

On the comparison of the SDS corresponding to the difficulty level assigned by each evaluator, a statistically significant difference (*P* < 0.001) was obtained (Table 8). Therefore, when the difficulty level based on the PSCES increases, the corresponding SDS increases significantly.

Pairwise comparison using Tukey's post hoc test confirmed that significant difference existed between individual difficulty levels when the SDS corresponding to the difficulty level (Table 9).

## 4. Discussion

The present study is aimed at determining whether the novel CBCT-based index could predict the surgical difficulty in maxillary sinus-augmentation procedures. The use of radiography-based indexes prior to the surgical procedure is known to reduce diagnostic errors [14]. Pederson was the first to propose a difficulty index for removal of the mandibular third molar on the basis of radiographic findings [15]. Although preoperative assessments of surgical difficulty are the most essential factor to be considered, it may be occasionally difficult to determine a single factor that increases the surgical difficulty because of the large anatomical variations among patients [16]. Therefore, some of the difficulty indices use a combination of radiographic and clinical parameters to determine the difficulty [17, 18].

The present study used an index purely based on imaging parameters. Although indices based on clinical and radiographic parameters are comprehensive, they tend to be time-consuming. Most radiography-based indices being used currently in dentistry are based on conventional radiographic techniques [19–21]. The index used in the present study evaluated surgical difficulty on the basis of CBCT findings. CBCT provides the advantage of a dimensionally accurate, multiplanar view of the surgical site [22], and CBCT-based indexes have been previously used for evaluating the dimensions and locations of periapical lesions [23]. Researchers have recently devised and validated a CBCT-based index for detecting osteoporosis in postmenopausal women [24], and the Impacted Canine Treatment Difficulty index (ICTD index) based on CBCT imaging has been recently used to assess the difficulty likely to be encountered during surgical and orthodontic alignment of impacted maxillary canine [25].

Implants play a vital role in modern-day dental practice, and they can now be placed at surgically modified sites, which were previously thought to be a contraindication for implant procedures [26]. The sinus-augmentation procedure is effective to gain bone height for implant placement in an atrophic posterior maxilla [8]. To the best of our knowledge, there is no CBCT-based index for predicting the surgical difficulty of sinus-augmentation procedures. In the present study, we attempted to develop and validate a novel difficulty index that can assist implantologists in thoroughly evaluating the site prior to the surgical procedure and predicting the difficulty of the surgical procedure. The PSCESs were compared and correlated with SDS to evaluate the ability of the index to predict surgical difficulty.

In the present study, the interrater agreement among the presurgical evaluators was 0.85. In a recent study, the interrater agreement for measurements of height and width of the maxilla using cross-sectional CBCT images was 0.75 [27]. This mild difference in interrater agreement values could be attributed to differences in the clinical specialties of evaluators between the two studies and the statistical method employed to estimate the agreement in the studies. The novel index used in the present study clearly defines the specific imaging plane that has to be used by the evaluator while assessing each parameter. Similar protocols were adopted in another study [27].

The novel CBCT-based index used in the present study included six parameters to determine the difficulty score. One of the primary parameters of the novel index was the thickness of the lateral wall of the sinus, which appears to influence the intactness of the sinus membrane [6]. In the present study, wall thickness of 3 to 5 mm was considered adequate. The thickness of the lateral wall was shown to increase from the first premolar region to the first molar region and decrease in the second molar region [28]. A recent study revealed that the thickness of the lateral wall of the maxillary sinus was lower in edentulous areas (1.31 ± 0.3 mm) [29], similar to the adequate range of the lateral wall thickness considered in the novel index. Greater thickness of the lateral wall has been known to make surgical procedures more difficult and longer [30]. This may be one of the factors responsible for the higher SDS in the present study. Another important factor influenced by lateral wall thickness is the choice of surgical instruments. A higher surgical difficulty is likely to be encountered if the Piezosurgical instruments are used to cut a thick lateral wall, since this approach would take a longer operating time than procedures performed with conventional surgical instruments [31].

The normal maxillary mucous membrane thickness has been reported to range from 0.3 to 0.9 mm, and mucosal swelling of more than 2 mm is considered pathologic in nature [31]. However, few other studies found average membrane thickness in the range of 1.60 ± 1.20 mm [32]. A recent meta-analysis stated that 3D imaging techniques tended to overestimate sinus membrane thickness by approximately 2.5-fold in comparison with the findings of histologic analyses [33]. Thus, there is no consensus regarding the average thickness of the sinus membrane and the threshold value above which the thickening can be considered pathologic [34]. In the novel index, a membrane thickness of 0.5 mm to 1.5 mm was considered adequate. Similar physiologic thickness values were observed in some other studies [35, 36]. A direct correlation has also been reported between the sinus membrane perforation rate and membrane thickness [37]. The chances of membrane perforation are the lowest when the membrane thickness is 1.5 mm [38]. Therefore, in the present study, patients with adequate thickness were likely to have a higher PSCESs and SDSs. The risk of membrane perforation during the sinus-augmentation procedure was also high if the sinus membrane of the maxillary sinus comes in contact with the root of the teeth adjacent to the edentulous space, thus making antrostomy at single-tooth edentulous spaces more difficult [39, 40]. Therefore, a higher difficulty score was assigned to a single-tooth edentulous area in the novel index.

Recent CBCT-based studies have revealed that the prevalence of septations in maxillary sinuses is as high as 22.5%-33% [41, 42]. Maxillary sinus septation tends to occur more frequently in edentulous subjects than in dentate subjects [43]. The incidence of sinus membrane perforation was 44.7% when the interfering septum was visualized in radiographic presurgical evaluations, whereas the incidence decreased to 2.4% when no radiographic evidence of sinus was found during presurgical evaluation [44]. The presence of septations on the maxillary floor necessitates the creation of two smaller windows on either side of the intervening septa, thus modifying the surgical technique [45]. If the septa are of a smaller size, a W-shaped hinge door preparation is recommended [46]. Septations in the transverse plane are associated with high levels of surgical difficulty and require a significant level of experience to manage, whereas septations in the coronal plane are associated with the least levels of surgical difficulty [44]. The novel CBCT index devised in the present study was based on several parameters in the region of interest (ROI). Similarly, a classification system for surgical difficulty in sinus-augmentation procedures based solely on the orientation and dimension of septa has been proposed in a previous study [47].

The alveolar antral artery is an important structure within the lateral maxillary sinus wall. Damage to this artery during surgical procedures or trauma can lead to profuse bleeding [48]. Haemorrhage associated with the alveolar antral artery is the second-most common intraoperative complication in sinus-augmentation procedures [49]. The alveolar antral artery was observed in all bodies in cadaveric studies but was detected in only 47%–67% of the patients in radiographic studies [50]. A possible explanation for this discrepancy could be that the small-diameter arteries (usually less than 0.5 mm) are not routinely detectable on CBCT or CT scans. Second, numerous alveolar antral arteries have subperiosteal pathways, preventing their detection on CBCT scans [51]. In the novel index, a higher difficulty score was assigned when radiographic evidence of the alveolar antral canal was evident. Studies have revealed that up to 20% of major bleeding events occurred due to accidental rupture of alveolar antral arteries, which considerably slowed the surgical procedure and thereby increased surgical difficulty [52].

Sinus pathologies and anatomical variations increase the risk of surgical complications during direct sinus-augmentation procedures [53–55]. A recent study showed that mucosal thickening (35.1%) is the most common radiographic finding, followed by sinus opacification (16.6%), polypoidal thickening (7.2%), and other pathologies (0.7%) [56]. Maxillary sinus pathologies were significantly higher in patients aged over 60 years [57, 58]. A recent study revealed that 45.1% of patients posted for maxillary sinus-augmentation surgery would require further consultation before the surgical procedure [59]. However, another recent study concluded that the presence of maxillary sinus pathology prior to surgery does not influence the survival rates of dental implants placed concurrently with sinus augmentation [60]. In the present study, a more balanced approach regarding sinus pathologies was considered while designing the novel index. Only specific pathologies were included in the novel index, based on the criteria mentioned in a previous study [13].

In the present study, the mean PSCES correlated positively with the mean SDS, and mean SDSs increased by a factor of 0.55 for every unit increase in mean PSCESs. These findings indicate that the PSCES is a reliable predictor of surgical difficulty. Although there are no similar indices or studies to compare these findings with, some radiographic index-based studies for predicting surgical difficulties have revealed good predictive values [61, 62]. This novel index can be used for specific clinical situations. Radiographic checklists or indices are particularly useful for inexperienced surgeons compared to experienced surgeons who are less likely to miss key preoperative findings [63]. In addition, radiographic indices are considered helpful for graduate students, trainees, and general dental practitioners in facilitating preoperative evaluation prior to performing new surgeries [64]. Checklists are particularly useful when multiple abnormalities are present in an image [65]. In a dental scenario, sinus pathology may receive all the attention of the surgeon who may then overlook the presence of alveolar antral artery. The use of an imaging index or checklist avoids such errors during presurgical evaluations.

### 4.1. Limitations and Scope for the Future Study

Although the present study offers some innovative insights, it also has a few shortcomings that should be addressed in future research. The present study did not include a control group of raters using conventional diagnostics. Therefore, we cannot convincingly state that CBCT imaging is a better predictor of the difficulty involved in sinus-augmentation procedures in comparison with conventional diagnostics. However, some recent studies have shown that CBCT provided better information of the sinus diagnostics and improved the prediction of possible complications in comparison with conventional diagnostics in sinus-augmentation procedures [66, 67].

Small sample size and moderate correlation are the other main limitations of the present study. A study involving larger sample size and multiple raters is recommended to reinforce the validity and reliability of the novel index. The novel index described in the present study uses parameters that are primarily concentrated at the ROI. Certain patient and surgeon factors also contributed to the overall difficulty of the cases. Future studies should aim to develop a comprehensive index that can take into consideration several parameters like the surgeon's experience, choice of surgical instruments, patient cooperation, smoking habits, and patient's dental health. Surgeons with inadequate experience in sinus augmentation may cause sinus membrane perforation more frequently during the procedure [68]. The type of equipment also influences the outcomes of sinus-augmentation procedures. A recent study used a specially designed bur that could create viscoelastic deformation of the bone at the augmentation site, resulting in lower incidence of Schneiderian membrane perforation [68]. Another study compared the outcomes of sinus-augmentation procedures using a surgical burr with a Piezotome [69]. Some of these factors should be considered while designing parameters for a clinic-radiographic index in the future.

## 5. Conclusions

The results of the present study suggest that the novel CBCT-based index can predict the surgical difficulty in maxillary sinus-augmentation procedures. We recommend the use of such novel indexes in radiographic evaluation of surgical sites as a standard protocol for comprehensive evaluation of direct sinus-augmentation procedures and forecasting the difficulty of the procedures.

## Figures and Tables

**Table 1:** The novel CBCT index with difficulty scores assigned to each parameter.

Parameter assessed	CBCT findings	Difficulty scores (DS)
(1) Thickness of the lateral window	Adequate (3–5 mm)	0
	Inadequate OR excessively thick	1
^∗^To be measured at the region of interest (ROI) in the coronal CBCT section		
(2) Thickness of the sinus membrane	Adequate (0.5–1.5 mm)	0
	Inadequate OR thick membrane	1
^∗^To be evaluated in the coronal section at the ROI ^∗^Sinus membrane thickness of more than 2 mm needs further consultation		
(3) Presence of sinus septations	Absent	0
	Present	1
^∗^To be evaluated at the ROI by using axial CBCT sections and further confirmed using coronal and sagittal sections		
(4) Relationship of sinus membrane with the roots of the adjacent teeth	Osteotomy at sites involving more than one missing tooth	0
	Osteotomy at sites involving one missing tooth	1
^∗^To be evaluated at the ROI by using sagittal sections		
(5) Radiographic presence of alveolar antral artery	Absent	0
	Present	1
^∗^Present implies visibility of the canal on the coronal CBCT section		
(6) Sinus pathologies	Absent	0
	Present	1
^∗^Sinus pathologies include the following: (a) Mucosal thickening of less than 2 mm with no current symptoms of sinusitis (b) Mucous-retention cyst filling less than 75% of the sinus (c) Bony oroantral communication masked by soft tissue. ^∗^Apart from the above-mentioned conditions, all other pathologies should be referred for additional presurgical evaluation. They should be only considered for augmentation once the pathology has resolved		
Presurgical CBCT evaluation score (PSCES)		

**Table 2:** The intrasurgical parameters and scoring pattern in the SDS checklist.

Intrasurgical parameter	Findings	Difficulty score (DS)
1. Operating time	Within 120 min	0
	More than 120 min	1

2. Perforation of the sinus membrane	Occurred	1
	Did not occur	0

3. Bleeding from alveolar antral artery	Occurred	1
	Did not occur	0

4. Damage or injury of adjacent teeth, fracture, fenestration, dehiscence, or perforation of alveolar bone, improper positioning or angulation of the fixture, and obstruction of the osteomeatal complex	Occurred	1
	Did not occur	0

**Table 3:** The postsurgical parameters and scoring pattern in the SDS checklist.

Postsurgical parameters	Findings	Difficulty score (DS)
1. Postoperative complications include pain, swelling, edema, infection of the surgical site and sinus, sinusitis, bone resorption, bleeding, oral and nasal ecchymosis and haematoma (haemosinus), emphysema, wound dehiscence, incisional breakdown, the loss of graft, dislocation, oroantral fistula, and temporary or permanent palatal numbness	Occurred	1
	Did not occur	0
Surgical difficulty score (SDS)		

**Table 4:** Interrater agreement of the PSCESs among the 5 presurgical evaluators.

	No. of raters	Fleiss' kappa	SE	95% CI
PSCES	5	0.85	0.03	0.80 to 0.91

**Table 5:** Interrater agreement of the SDS among performer and observers of the sinus-augmentation procedures.

SDS	Kappa value
Performer vs. observer 1	0.82
Performer vs. observer 2	0.82
Observer 1 vs. observer 2	0.64

**Table 6:** Correlation between PSCES and SDS of the performer.

	Performed SDS	
	*r*	*P* value
PSCES	0.76	<0.001^∗^
PSCES	0.71	<0.001^∗^
PSCES	0.68	<0.001^∗^
PSCES	0.71	<0.001^∗^
PSCES	0.68	<0.001^∗^
Average PSCES	0.72	<0.001^∗^

**Table 7:** Linear regression between independent variable PSCES and dependent variable SDS.

	Unstandardized coefficients		Standardized coefficients	*t*	*P* value	95.0% confidence interval for B	
	*B*	Std. error	Beta			Lower bound	Upper bound
(Constant)	1.10	0.26		4.22	<0.001^∗^	0.57	1.64
PSCES evaluator 1	0.53	0.09	0.76	6.13	<0.001^∗^	0.36	0.71
(Constant)	1.11	0.29		3.78	0.001^∗^	0.51	1.71
PSCES evaluator 2	0.57	0.11	0.71	5.32	<0.001^∗^	0.35	0.79
(Constant)	1.15	0.31		3.73	0.001^∗^	0.52	1.78
PSCES evaluator 3	0.54	0.11	0.68	4.90	<0.001^∗^	0.32	0.77
(Constant)	1.11	0.29		3.78	0.001^∗^	0.51	1.71
PSCES evaluator 4	0.57	0.11	0.71	5.32	<0.001^∗^	0.35	0.79
(Constant)	1.16	0.31		3.75	0.001^∗^	0.52	1.79
PSCES evaluator 5	0.53	0.11	0.68	4.89	<0.001^∗^	0.31	0.76
(Constant)	1.08	0.29		3.71	0.001^∗^	0.48	1.68
Average PSCES	0.57	0.10	0.72	5.48	<0.001^∗^	0.36	0.78

Dependent variable: mean SDS. PSCES 1: −*F*(1, 28) = 37.62, *P* < 0.001, *r*^2^ = 0.57. PSCES 2: −*F*(1, 28) = 28.29, *P* < 0.001, *r*^2^ = 0.50. PSCES 3: −*F*(1, 28) = 24.04, *P* < 0.001, *r*^2^ = 0.46. PSCES 4: −*F*(1, 28) = 28.29, *P* < 0.001, *r*^2^ = 0.50. PSCES 5: −*F*(1, 28) = 23.95, *P* < 0.001, *r*^2^ = 0.46. PSCES Avg: −*F*(1, 28) = 30.05, *P* < 0.001, *r*^2^ = 0.52. ^∗^*P* < 0.05, statistically significant; *P* > 0.05, nonsignificant (NS).

**Table 8:** Overall comparison of SDS corresponding to the difficulty level assigned by each evaluator.

	Difficulty level	*N*	Mean	Std. deviation	Minimum	Maximum	ANOVA	
							*F*	*P* value
Evaluator 1	Minimal difficult	13S	1.31	0.48	1	2	60.77	<0.001^∗^
	Moderate difficult	13	3.00	0.58	2	4		
	Difficult	4	4.25	0.50	4	5		

Evaluator 2	Minimal difficult	13	1.31	0.48	1	2	60.77	<0.001^∗^
	Moderate difficult	13	3.00	0.58	2	4		
	Difficult	4	4.25	0.50	4	5		

Evaluator 3	Minimal difficult	13	1.31	0.48	1	2	39.77	<0.001^∗^
	Moderate difficult	14	3.14	0.77	2	5		
	Difficult	3	4.00	0.00	4	4		

Evaluator 4	Minimal difficult	13	1.31	0.48	1	2	60.77	<0.001^∗^
	Moderate difficult	13	3.00	0.58	2	4		
	Difficult	4	4.25	0.50	4	5		

Evaluator 5	Minimal difficult	13	1.31	0.48	1	2	34.49	<0.001^∗^
	Moderate difficult	12	3.17	0.84	2	5		
	Difficult	5	3.60	0.55	3	4		

^∗^ *P* < 0.05, statistically significant; *P* > 0.05, nonsignificant (NS).

**Table 9:** Pairwise comparison of SDS corresponding to the difficulty level assigned by each evaluator.

	Difficulty level (I)	Difficulty level (J)	Mean difference (I-J)	Std. error	*P* value	95% confidence interval	
						Lower bound	Upper bound
Evaluator 1	1	2	-1.69	0.21	<0.001^∗^	-2.21	-1.18
		3	-2.94	0.30	<0.001^∗^	-3.69	-2.19
	2	3	-1.25	0.30	0.001^∗^	-2.00	-0.50

Evaluator 2	1	2	-1.69	0.21	<0.001^∗^	-2.21	-1.18
		3	-2.94	0.30	<0.001^∗^	-3.69	-2.19
	2	3	-1.25	0.30	0.001^∗^	-2.00	-0.50

Evaluator 3	1	2	-1.84	0.24	<0.001^∗^	-2.43	-1.24
		3	-2.69	0.40	<0.001^∗^	-3.68	-1.70
	2	3	-0.86	0.40	0.10 (NS)	-1.84	0.13

Evaluator 4	1	2	-1.69	0.21	<0.001^∗^	-2.21	-1.18
		3	-2.94	0.30	<0.001^∗^	-3.69	-2.19
	2	3	-1.25	0.30	0.001^∗^	-2.00	-0.50

Evaluator 5	1	2	-1.86	0.26	<0.001^∗^	-2.51	-1.21
		3	-2.29	0.35	<0.001^∗^	-3.15	-1.44
	2	3	-0.43	0.35	0.44 (NS)	-1.30	0.43

Tukey's post hoc test. ^∗^*P* < 0.05, statistically significant; *P* > 0.05, nonsignificant (NS).

## Data Availability

The data is available at doi:10.6084/m9.figshare.14637261.v1.

## Abbreviations

CBCT: Cone-beam computerized tomography
DS: Difficulty score
PSCES: Presurgical CBCT evaluation score
SDS: Surgical difficulty score.
